# Per- and polyfluoroalkyl substances (PFAS) exposure profiles for pregnant women living in urban, rural, and remote areas of Suriname, South America suggest higher serum concentrations in remote rainforest communities

**DOI:** 10.1038/s41370-026-00950-y

**Published:** 2026-07-23

**Authors:** Jenna K. Honan, Firoz Abdoel Wahid, Anisma Gokoel, Maureen Y. Lichtveld, Wilco Zijlmans, Hannah H. Covert, Ashna D. Hindori-Mohangoo, Jimmy Roosblad, John Codrington, Carin A. Huset, Kitrina M. Barry, Lisa A. Peterson, Karin R. Vevang, Megan E. McCabe, Jeffrey K. Wickliffe

**Affiliations:** 1 Department of Environmental Health Sciences, School of Public Health, University of Alabama at Birmingham, Birmingham, AL, USA.; 2 Department of Environmental and Occupational Health, School of Public Health, University of Pittsburgh, Pittsburgh, PA, USA.; 3 Faculty of Medical Sciences, Anton de Kom University, Paramaribo, Suriname.; 4 Foundation for Perinatal Interventions and Research in Suriname (Perisur), Paramaribo, Suriname.; 5 Clinical Chemistry Laboratory, Academic Hospital Paramaribo, Paramaribo, Suriname.; 6 Minnesota Department of Health, Saint Paul, MN, USA.; 7 Masonic Cancer Center and Division of Environmental Health Sciences, School of Public Health, University of Minnesota, Minneapolis, MN, USA.; 8 Department of Biostatistics, School of Public Health, University of Alabama at Birmingham, Birmingham, AL, USA.

**Keywords:** Maternal health, Persistent organic pollutants (POPs), Global health, Environmental pollution, Biomonitoring

## Abstract

**BACKGROUND:**

Per- and polyfluoroalkyl substances (PFAS) are a diverse group of organofluorine compounds used in industrial and consumer applications. The primary route of human exposure to PFAS is ingestion of contaminated food and drinking water, representing a significant and sometimes unavoidable exposure for many populations. Ecotoxicological and epidemiological studies have established associations between PFAS exposures and adverse health effects. Fetal and childhood development is a particularly susceptible window of exposure.

**OBJECTIVE:**

We characterized PFAS exposures for pregnant women in Suriname who identified as Indigenous or Tribal Peoples, and we examined regional variations in exposure patterns between urban, rural, and remote populations.

**METHODS:**

We measured 24 PFAS analytes in the blood serum of 100 pregnant Tribal and Indigenous women living in Suriname. Samples were collected from December 2016 to July 2019 and were purposively selected for laboratory analysis. We assessed differences in serum concentrations visually and statistically by location and ethnicity.

**RESULTS:**

Ten unique PFAS were detected in at least one sample. The median total PFAS across all participants was 1.05 ng/mL (IQR: 0.73–1.45 ng/mL). The most frequently detected PFAS were PFOS (DF = 100%, median concentration $(\tilde{x})=0.59\;\text{ng/mL}$), PFOA (DF = 86%, $\tilde{x}=0.15\;\text{ng/mL}$), PFNA (DF = 84%, $\tilde{x}=0.09\;\text{ng/mL}$), PFUnDA (DF = 72%, $\tilde{x}=0.09\;\text{ng/mL}$), and PFHxS (DF = 71%, $\tilde{x}=0.06\;\text{ng/mL}$). Median total PFAS concentrations were highest for participants in the remote interior district, although differences among the districts were not statistically significant (*p* = 0.37) and should be interpreted cautiously due to limited statistical power. Indigenous Peoples tended to have higher total PFAS in their blood serum ($\tilde{x}=1.25\;\text{ng/mL}$) compared to Tribal Peoples ($\tilde{x}=0.84\;\text{ng/mL}$, *p* < 0.01), although we did not assess potential confounding variables.

**SIGNIFICANCE:**

Our analysis revealed mostly long-chain legacy PFAS with possibly different exposure risks for secluded interior communities compared to urban-coastal populations, indicating a need for contaminant source assessments.

**IMPACT:**

Our study adds to the growing literature around PFAS exposures for people living in remote areas. Suriname aims to reduce environmental contamination from persistent organic pollutants through the Stockholm Treaty. Our study provides data needed for policymakers and communities to make informed decisions about their environmental health.

## INTRODUCTION

Per- and polyfluoroalkyl substances (PFAS) are a diverse group of organofluorine compounds that have been widely used in modern societies over the last 85 years, with both industrial and consumer applications [1]. While PFAS are known environmental contaminants in urbanized settings, these synthetic chemicals are increasingly detected in rural and remote environments, including those with no known proximal sources. While most of the secluded communities with measured PFAS live in arctic, subarctic, or other cold climates [2], few studies have evaluated PFAS exposures in tropical remote regions. Suriname, a country located in northern South America near the equator, presents a unique environmental and epidemiological context for investigating PFAS exposure in urban, rural, and remote areas.

PFAS encompass thousands of distinct chemical compounds, including perfluorooctanoic acid (PFOA), perfluorononanoic acid (PFNA), perfluorohexanesulfonic acid (PFHxS), and perfluorooctanesulfonic acid (PFOS). These compounds are characterized by their unique molecular structure, with the strong carbon-fluorine bonds providing desirable qualities for use in consumer or commercial products, such as water and grease resistance, thermal stability, and surfactant properties [3]. The same sturdy structure that provides exceptional stability also resists microbial, chemical, and physical degradation processes [3, 4], and the resulting environmental persistence has earned PFAS an apt nickname: “forever chemicals.” These chemicals are ubiquitous, highly mobile, and can remain in the environment for hundreds of years, creating a situation of widespread environmental contamination and regular human exposure [4–6]. The recalcitrant nature of these compounds in both environmental and biological systems has raised global public health concerns and garnered substantial environmental health research attention [7]. However, to date, only a few PFAS chemicals have been scientifically evaluated for adverse human health outcomes related to exposure [1], and existing population-level studies have been heavily focused on urban areas.

Suriname has experienced a geographical gradient of industrialization, with urbanization occurring primarily along the northern coastline. Extensive rainforests comprise the remote southern interior district of Sipaliwini, which covers nearly 80% of the country by land but contains only around 5% of the country’s population [8, 9]. Northern coastal districts like Paramaribo and Nickerie include both cities and commercial agricultural sites and house approximately 90% of the Surinamese people [8–10]. Other districts with lower population density, such as Para, are predominantly rural but maintain transportation access to urban areas. The northern districts represent areas with greater access to commercial goods and services, imported products, and processed foods [9, 10]. The country’s economy is reliant on the export of gold, oil, and agricultural products [11].

While many of the larger urban cities in Suriname, such as Paramaribo or Nieuw Nickerie, tend to be more demographically diverse, smaller communities exist in the Amazonian interior part of Suriname, including Tribal Peoples, who are descendants of former African slaves, as well as native Indigenous Peoples, whose ancestors have lived in the region for thousands of years. Indigenous Peoples include the Kalina (Caribs), Lokono (Arawak), Tiriyó (Trio), and Wayana, and Tribal Peoples include the Aluku (Boni), Kwinti, Matawai, Ndyuka (Aukan), Paramaka, and Saramaka. Although census numbers may undercount both Tribal and Indigenous Peoples, Indigenous communities in Suriname comprise approximately 3–4% of the population, while estimates for Tribal Peoples are between 15 and 22% of the population [10, 12].

Indigenous and Tribal Peoples inhabit over 200 villages across Suriname [12]. Some of these smaller communities in districts like Para reside inland in the tropical forests with limited but available access to the cities. Other interior communities, such as those in Sipaliwini, are secluded and tend to maintain traditional subsistence means of living with locally harvested foods, limited consumerism, and reliance on nearby surface waters or ground-water wells for drinking water. This dichotomy between the urbanized areas, with widespread availability and use of PFAS-containing materials, and the remote areas, where communities often follow natural and subsistence lifestyles, provides real-world study conditions for examining the relative contributions of different environmental exposure pathways.

Human exposure to PFAS occurs through multiple pathways, reflecting the omnipresent nature of these compounds, especially in industrialized settings. Consumer products containing PFAS are pervasive in modernized societies and include non-stick cookware, stain-resistant coatings on furniture, carpets, rugs, and other home items, water-repellent clothing and outdoor recreation gear, cleaning products, and various personal care products such as cosmetics, dental floss, and eyeglass cleaners [3, 13–16]. Industrial applications include PFAS-containing paints, varnishes, sealants, and pesticides [3, 15, 17]. Firefighters use specialized aqueous film-forming foams (AFFF) that contain PFAS to extinguish and prevent the spread of flames, and PFAS are also present in their personal protective equipment [18]. PFAS are in many of the materials that come into contact with foods, particularly processed foods, such as containers, wrappers, boxes, “microwave-safe” bags, and paper plates and cups [19]. Recent changes to regulations in some parts of the world seek to prevent their continued use in food packaging [20, 21].

The primary route of exposure to PFAS is through ingestion via consumption of contaminated food and drinking water [4], representing a significant and sometimes unavoidable exposure for many populations. Excretion of these compounds is slow relative to many other environmental chemicals, as they tend to have long half-lives in the human body [22]. PFAS can bioaccumulate, and when people eat fish or other aquatic animals living in PFAS-contaminated waters, biomagnification can occur, substantially elevating body burdens of subsistence fishing communities [6, 23]. Ingestion of crops or livestock grown on or raised near areas where PFAS are released into the environment, either purposefully or accidentally, or where biosolid fertilizers that contain PFAS are used, can further exacerbate exposure [19, 24, 25]. Even low-dose and short-term exposures, such as incidental ingestion of contaminated soil or dust or occasional recreational activities in polluted environments, can contribute to the cumulative PFAS burden in people [26, 27].

Ecotoxicological and epidemiological studies have established associations between PFAS exposures and adverse health effects across numerous organ systems. Endocrine disruption, reproductive and developmental disorders, metabolic dysregulation, kidney and testicular cancers, elevated low-density lipoprotein cholesterol, and hepatotoxicity have all been attributed to elevated PFAS exposure [3, 28–33]. These outcomes can occur after exposure to environmental concentrations in parts per trillion.

The implications of PFAS exposure during pregnancy elicit particular concern due to the growing evidence of adverse maternal and fetal health effects. As is the case for many environmental exposures, fetal and childhood development appears to be a particularly susceptible window of exposure [34]. PFAS have been associated with increased risks of preeclampsia and gestational hypertension, both of which pose risks to both mother and fetus [35]. The health implications for children who were exposed to PFAS during pregnancy are increasingly documented in studies that show associations with liver injury, metabolic dysregulation, and possible neurodevelopmental effects [36–39]. Small reductions in birth weight were seen for infants born to mothers with elevated blood serum PFAS concentrations, indicating an effect on fetal growth and development [40]. Other studies have found associations between PFAS in infants’ blood serum and attenuated immunological responses to later childhood vaccines [41]. Emerging research indicates children who were exposed to PFAS in utero may experience issues with neurological development, although the magnitude and persistence of these outcomes are currently unclear [37]. Presence of PFAS in the cord blood, placenta, and breastmilk of exposed mothers underscores the vulnerability of developing fetuses with the likelihood of continued exposure into infancy [42, 43].

The primary objective of our investigation was to characterize PFAS exposures for pregnant women in Suriname, especially in Indigenous and Tribal communities, and to examine regional variations in exposure patterns between urban, rural, and remote populations. The extent of PFAS contamination in the country remains almost entirely undefined. One study, published in 2020 by Pinas et al. [44], developed a preliminary inventory of PFOS sources used in Suriname as an initial step toward meeting the Stockholm Convention treaty, which requires the protection of people and ecosystems from persistent organic pollutants (POPs). However, a critical question remains about the likely differential exposures between interior and coastal districts given the dissimilar lifestyles, access to resources, and environments, especially for the remote communities.

Results from our pilot study provide PFAS exposure profiles for pregnant Tribal and Indigenous women living in distinct communities across Suriname. Given the metropolitan settings in which these compounds are commonly used, we expected the highest blood serum concentrations paired with higher detection frequencies to be from participants living in northern urban areas and the lowest concentrations and detection frequencies from those residing in remote interior regions. Our study establishes baseline PFAS biomarker data that can be used to identify potential exposure pathways and sources of pollution in Suriname or to evaluate changes in exposure over time. The data presented in this study are relevant for health risk assessments, intervention planning and implementation, environmental health advocacy, and community decision-making.

## METHODS

### Study design and recruitment

As a parent study, the Caribbean Consortium for Research in Environmental and Occupational Health (CCREOH) conducted a longitudinal cohort study to collect whole blood and serum samples from 1143 pregnant women in their late first, early second, or third trimester. To be included in the CCREOH study, participants had to be at least 16 years of age, pregnant and carrying a single fetus, speak Dutch, Saramaccan, Sarnami, Sranan Tongo, or Trio, and plan to deliver their baby at one of the clinical or midwifery recruitment sites. For women aged 16 and 17, we asked for assent from the participants and for consent from their parents, with assent asked in addition to the consent form. Recruitment occurred at the clinics of the Regional Health Services, at hospitals in Paramaribo and Nickerie, and through the Medical Mission Primary Health Care Suriname for participants living in the interior. Samples were collected between December 2016 and July 2019. Zijlmans et al. [10] further describe methodologies employed by the CCREOH parent project.

To quantify PFAS blood serum concentrations for our study, we selected 100 samples from the CCREOH participants (*n* = 100/1143, 8.7%). The subsample included only women who self-identified as being either Tribal or Indigenous Peoples at intake. In the CCREOH study, 271 (23.7%) participants identified as Tribal Peoples, 155 (13.6%) identified as Indigenous Peoples, while the remaining women (*n* = 717) were Creole, Hindustani, Javanese, mixed race, or another race. From the 426 available serum samples from Tribal or Indigenous women, we chose participants (*n* = 100/426, 23.5%) to represent the range of districts present in the CCREOH study, including the densely populated capital (Paramaribo district), the agricultural districts in the north (Nickerie and Para districts), and the secluded interior rainforest (Sipaliwini district).

Half of the samples (*n* = 50) were from Surinamese women who accessed care across the northern districts (i.e., Paramaribo, Nickerie, and Para), and half (*n* = 50) were from women living in the interior Sipaliwini district, although one participant was later reassigned from the northern districts group to the Sipaliwini district group. Some participants had a different district of residence than the district where they received care, although this was uncommon. For example, residents of the Brokopondo district who received care in Para would be included in the Para district subgroup. Although the CCREOH study collected samples from participants at multiple timepoints, for this analysis, we used only one sample from one timepoint for each included participant. The majority of the selected samples were collected during the initial CCREOH visit. We did not use any other selection criteria beyond race/ethnicity and location of care.

All aspects of this study design were approved by the Institutional Review Boards at the Medical Ethical Commission of Suriname’s Ministry of Health, Paramaribo, Suriname (approval ID: VG 023-14) and Tulane University, New Orleans, Louisiana, USA (approval number: 839093).

### Data collection and sample storage

During the initial visit of the CCREOH study, participants provided whole blood and serum samples, as well as demographic information including place of residence, district where healthcare was accessed, ethnicity, education level, monthly household income, and age, among other information and biological samples (e.g., urine or hair). Some participants provided additional biological samples at follow-up visits. The blood samples were collected in serum-separator vacutainers at the recruitment site hospitals and clinics before being delivered to the Academic Hospital Paramaribo clinical laboratory. Samples from participants in the remote areas were collected and stored for up to 48 h at 4 °C prior to delivery. For long-term sample storage, blood, plasma, and serum samples were aliquoted into 2 mL freezer tubes and frozen at −80 °C. Samples were shipped to the United States and maintained at −80 °C. The 100 serum samples selected for supplemental PFAS analysis were then shipped to an external laboratory. All samples were shipped by priority on dry ice and did not go through any freeze-thaw cycles.

### Laboratory analysis

The Minnesota Department of Health Environmental Laboratory quantified 24 distinct PFAS chemicals in our study samples as part of the National Institutes of Environmental Health Sciences Human Health Exposure Analysis Resource (HHEAR) program. The compounds included both legacy (i.e., typically long-chain and generally phased out of production in many countries) and emerging (i.e., usually short-chain and designed as replacements for legacy chemicals) PFAS. The laboratory participates in the Centre de Toxicologie du Québec Arctic Monitoring and Assessment Programme (CTQ AMAP) Ring Test for Persistent Organic Pollutants in Human Serum.

The serum analysis was performed through protein precipitation and quantified using liquid chromatography tandem mass spectrometry (LC/MS/MS). A stable isotope-labeled internal standard solution and acetonitrile were added to a 200 μL aliquot of serum in microcentrifuge tubes. The tubes were mixed, centrifuged, and an aliquot of the supernatant was transferred to a 96-well plate for concentration. After concentration, the sample was analyzed by LC/MS/MS (Shimadzu Prominence coupled to AB Sciex 5500Q) using electrospray ionization with multiple reaction monitoring (MRM) and retention time windows. Charcoal-stripped bovine calf serum was used to prepare matrix-matched calibration curves, and quantitation was performed via isotope dilution. A minimum of two MRM transitions were monitored for each analyte (when possible), and unknowns were verified through retention time and ion ratios matching with the calibration curve.

A minimum of three quality control (QC) samples were run with each batch of 20 unknowns: (1) a method blank to assess laboratory contamination and background levels, and (2) low and (3) high levels of PFAS in human serum to verify method accuracy and precision across the analytical range. An additional QC was run if an unknown sample required dilution, either due to a low volume of sample or an initial concentration higher than the calibration curve allowed. Batches with QC recovery outside of 70–130% were reanalyzed. In cases where a QC failure could not be resolved through reinjection/reanalysis, the resulting unknown data were qualified to reflect this result.

The method detection limit (MDL) was calculated during validation using the standard deviation of replicates of blanks and low-level calibration curve points over several days. The MDL for this method ranges from 0.003 to 0.066 ng/mL. For a 200 μL sample volume, reporting levels were 0.025 ng/mL for perfluorobutanesulfonic acid (PFBS), PFHxS, PFOS, and 4,8-dioxa-3H-perfluorononanoic acid (ADONA). Reporting levels of 0.05 ng/mL were established for perfluorohexanoic acid (PFHxA), perfluoroheptanoic acid (PFHpA), PFOA, PFNA, perfluorodecanoic acid (PFDA), perfluoroundecanoic acid (PFUnDA), perfluorododecanoic acid (PFDoDA), perfluoroheptanesulfonic acid (PFHpS), perfluorodecane sulfonic acid (PFDS), perfluorooctane sulfonamide (FOSA), 4:2 fluorotelomer sulfonate (4:2FtS), 8:2 fluorotelomer sulfonate (8:2FtS), hexafluoropropylene oxide dimer acid (GenX), 9-chlorohexadecafluoro-3-oxanonane-1-sulfonic acid (9Cl-PF3ONS), and 11-chloroeicosafluoro-3-oxaundecane-1-sulfonic acid (11Cl-PF3OUdS). Higher reporting levels of 0.1 ng/mL were designated for perfluorobutanoic acid (PFBA), perfluoropentanoic acid (PFPeA), N-methyl perfluorooctane sulfonamidoacetic acid (NMeFOSAA), N-ethyl perfluorooctane sulfonamidoacetic acid (EtFOSAA), and 6:2 fluorotelomer sulfonate (6:2FtS).

### Geographical analysis

Using the location of residence for each participant, we evaluated blood serum PFAS concentrations across districts and communities. We categorized communities as primarily Indigenous Peoples, primarily Tribal Peoples, or a mix of both. While many communities in Suriname, particularly those situated in the interior, can be categorized as predominantly Indigenous or Tribal Peoples, there may still be some mixing of subpopulations within each location. We then visually assessed the geographical distribution of total PFAS concentrations across the country.

### Statistical analysis

We calculated appropriate descriptive statistics (e.g., median, mean, interquartile range (IQR) as the 25th and 75th percentiles, standard deviation (SD), and detection frequency (DF) as the number of samples with detectable PFAS out of the 100 total study samples) for serum PFAS concentrations and participant demographic information. We calculated total PFAS per sample as the sum of each individual PFAS concentration, treating non-detects as 0 ng/mL. We evaluated the normality of the data visually and by comparing the mean and median values. Prior to statistical tests, we log-transformed skewed variables [45]. We summarized continuous variables using the arithmetic mean and SD if approximately normally distributed; otherwise, we used the geometric mean or median and IQR. Categorical variables were summarized using frequencies and percentages.

Serum concentration distributions were assessed for differences by district, by ethnicity, and by district and ethnicity together. For normally distributed data, we used parametric statistical tests (i.e., *t*-test, ANOVA) with a significance threshold of α = 0.05 to indicate differences by group. For variables where the distribution remained notably skewed after log-transformation, appropriate non-parametric equivalents (i.e., Wilcoxon Rank-Sum Test, Kruskal–Wallis) were used instead. A post hoc power test for detecting differences among districts in total PFAS concentration using ANOVA with an effect size (*f*) of 0.18 (derived from *η*^2^ = 0.03), an alpha (*α*) of 0.05, and a total sample size (*n*) of 100 among the four districts indicated limited power (<30%), and therefore our results should be interpreted as preliminary.

Non-detects were excluded from individual PFAS analyses, although we did conduct a sensitivity analysis using multiple imputation by chained equations to evaluate the effect of their exclusion. We multiply imputed the data only for a subset of the PFAS with less than 30% missing (i.e., non-detect) values (PFOA, PFNA, PFDA, PFUnDA, PFHxS, and PFOS) using concentrations from the other detected individual PFAS, participant ethnicity, and district of care [46, 47]. We then recalculated total PFAS sample concentrations by summing the six imputed and four non-imputed datasets for the same 10 PFAS included in the original total PFAS calculations. Non-detects were the only type of missing data for the serum concentrations. We did not adjust for multiple comparisons due to the exploratory nature of this work.

All statistical and graphical analyses were conducted using Prism (GraphPad, version 10.6.1 (892)) or RStudio (version 2026.01.0 + 392) with R software (version 4.5.2). De-identified data and code used for data analyses are available upon request.

## RESULTS

We measured 24 PFAS analytes in the blood serum of 100 pregnant Tribal and Indigenous women living in Suriname (Table 1), with 25 participants from urban Paramaribo, 11 from rural Nickerie, 13 from rural Para, and 51 from the remote interior Sipaliwini. The mean age of participants at baseline was 26.0 years (SD: 6.6 years, range: 16–48 years). The majority of participants identified as Indigenous (56%, *n* = 56), and the remainder (44%, *n* = 44) as Tribal Peoples. The two groups varied by district, with most of the participants within our sample who identified as Tribal Peoples living in Paramaribo (*n* = 17/44), followed by Sipaliwini (*n* = 16/44) and Para (*n* = 11/44), while Indigenous participants were primarily from Sipaliwini (*n* = 35/56), followed by Nickerie (*n* = 11/56), Paramaribo (*n* = 8/56), and Para (*n* = 2/56). None of the Tribal participants lived in Nickerie.

Tribal women tended to have higher formal education levels than did Indigenous women (*p* < 0.01) and were similarly more likely to have higher household incomes, although the difference was not statistically significant (*p* = 0.13) when stratified across multiple income categories. For both groups, most women had annual household incomes below $18,000 Surinamese dollars (SRD), although Indigenous women in our study were more likely to fall below this threshold (73%) than were Tribal women (48%, *p* = 0.04). The mean per capita income in Suriname for 2019 was $47,465 SRD [48, 49]. For about one-third of the women (31%), this was their first live birth, although a few had been previously pregnant. The correlation between gravidity and parity was higher for Tribal women (Pearson’s correlation coefficient (*r*)=0.95) than for Indigenous women (*r* = 0.86).

**Fig. 1:**
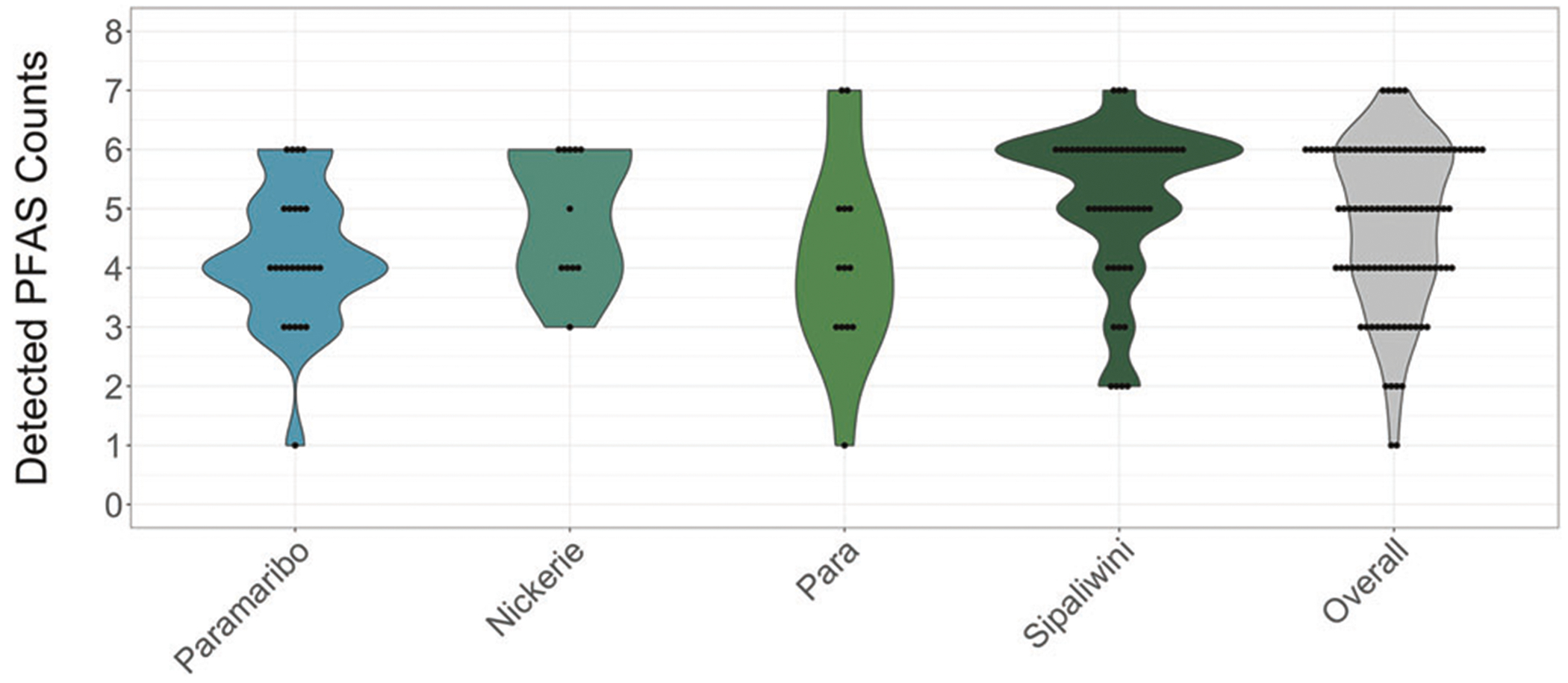
Frequency (counts) of unique PFAS compounds per blood serum sample by district. Participants from the interior district of Sipaliwini (*n* = 51) were most likely to have higher numbers of distinct PFAS types detected in their blood serum samples compared to Paramaribo (*n* = 25), Nickerie (*n* = 11), and Para (*n* = 13).

Of the 24 PFAS compounds analyzed, 10 (41.7%) were detected in at least one sample (Table 2). The most frequently detected PFAS were PFOS (DF = 100%), PFOA (DF = 86%), PFNA (DF = 84%), PFUnDA (DF = 72%), and PFHxS (DF = 71%), all of which are considered legacy compounds. Nine unique PFAS chemicals were detected in samples from participants corresponding to the interior district of Sipaliwini, the highest number of distinct PFAS compounds of the four districts (Table 2). Individuals in our study sample from Sipaliwini tended to have higher counts of unique PFAS types in their samples (median = 6 compounds, maximum = 7), followed by Nickerie (median = 5 compounds, maximum = 6) and Paramaribo (median = 4 compounds, maximum = 6, Fig. 1). Although Para participants tended to have lower counts of distinct PFAS detected per sample (median = 4 compounds), the maximum was 7 compounds, equal to that of Sipaliwini participants.

NMeFOSAA had the highest median concentration across all districts ($\tilde{x}=0.65\;\text{ng/mL}$), although it was only detected in a single serum sample from a participant in Para (Table 2). PFOS had a similar median concentration ($\tilde{x}=0.59\;\text{ng/mL}$) and was detected in all samples (*n* = 100/100). PFOA had a median concentration of 0.15 ng/mL, followed by PFDoDA ($\tilde{x}=0.10\;\text{ng/mL}$), PFUnDA ($\tilde{x}=0.09\;\text{ng/mL}$), PFNA ($\tilde{x}=0.09\;\text{ng/mL}$), PFDA ($\tilde{x}=0.08\;\text{ng/mL}$), PFHxS ($\tilde{x}=0.06\;\text{ng/mL}$), and PFHpS ($\tilde{x}=0.06\;\text{ng/mL}$). The median total PFAS concentration for all participants was 1.05 ng/mL (IQR: 0.73–1.45 ng/mL, range: 0.18–4.64 ng/mL, geomean: 1.03 ng/mL).

**Fig. 2:**
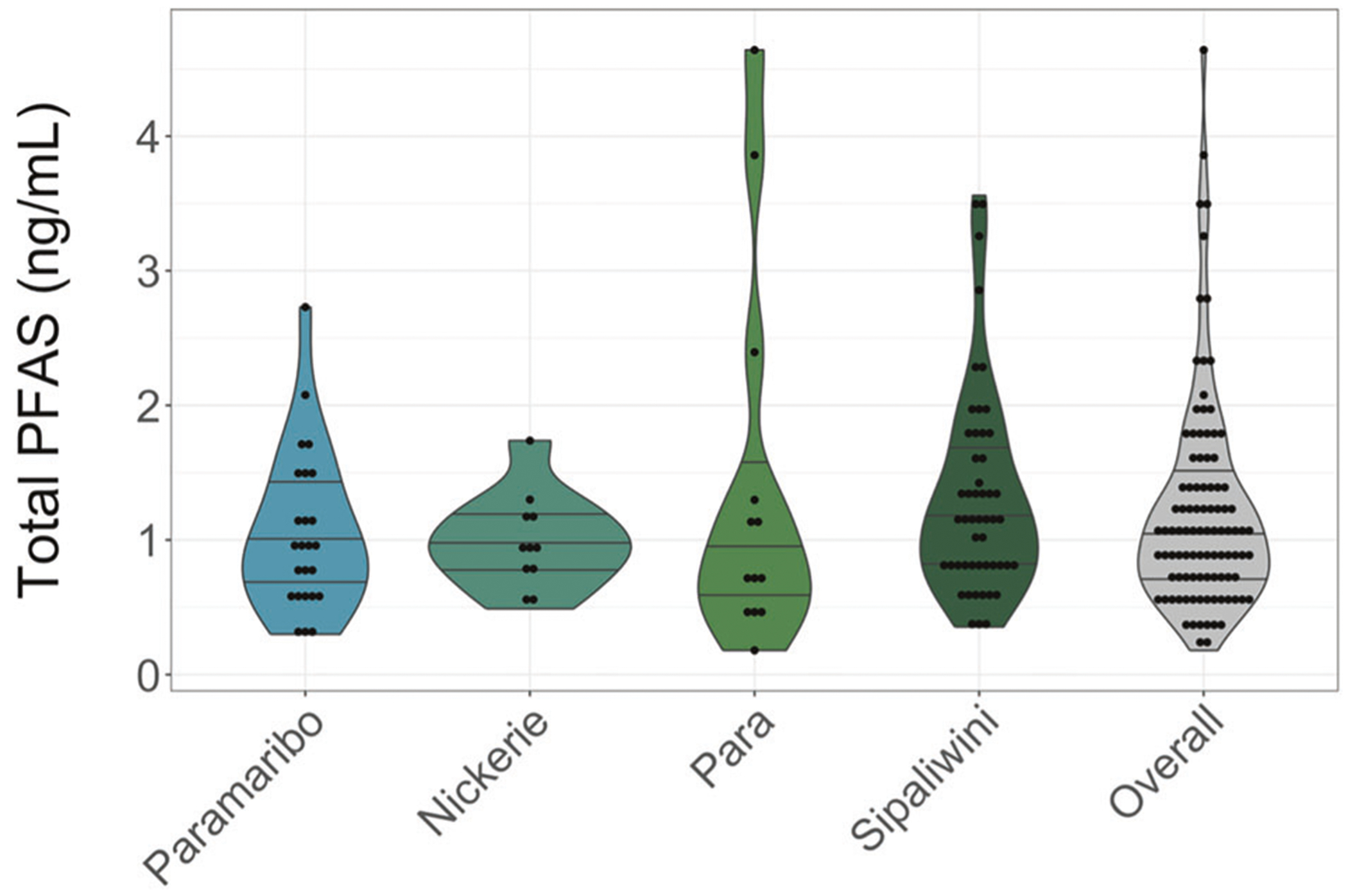
Distributions of total PFAS concentrations (ng/mL) in blood serum samples for each district. Participants from Sipaliwini had the highest median concentration of total PFAS (*n* = 51, $\tilde{x}=1.12\;\text{ng/mL}$), followed by Nickerie (*n* = 11, $\tilde{x}=1.00\;\text{ng/mL}$), Paramaribo (*n* = 25, $\tilde{x}=0.93\;\text{ng/mL}$), and Para (*n* = 13, $\tilde{x}=0.78\;\text{ng/mL}$). The lines represent the 25th, 50th, and 75th percentiles. Differences in log-transformed concentrations were not statistically significant (*p* > 0.05) among the districts.

**Fig. 3:**
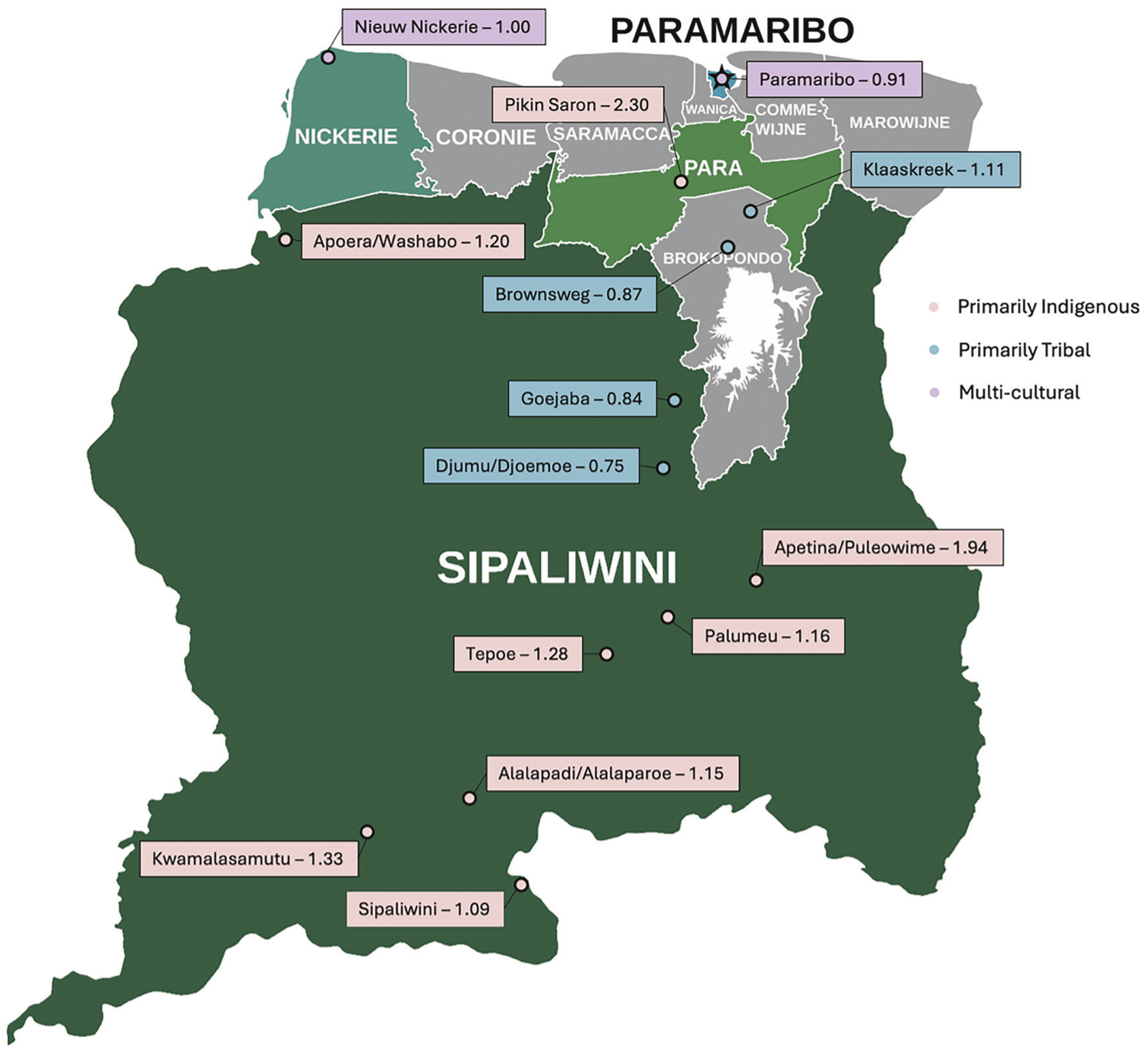
Median total PFAS concentrations (ng/mL) in blood serum samples by location of residence. For each participant in our study sample, we measured total PFAS concentrations in blood serum. This map shows cities, towns, and community locations of participants, with pink locations indicating primarily Indigenous Peoples, blue locations indicating primarily Tribal Peoples, and purple locations indicating multi-cultural areas, which include similar population rates of Tribal and Indigenous participants. However, pink locations may include Tribal participants, and blue locations may include Indigenous participants. Colored regions represent the four districts where participants accessed healthcare.

Participants from Sipaliwini (*n* = 51) had the highest median concentration of total PFAS ($\tilde{x}=1.12\;\text{ng/mL}$), followed by Nickerie (*n* = 11, $\tilde{x}=1.00\;\text{ng/mL}$), Paramaribo (*n* = 25, $\tilde{x}=0.93\;\text{ng/mL}$), and Para (*n* = 13, $\tilde{x}=0.78\;\text{ng/mL}$, Table 2, Fig. 2). Concentrations did not differ significantly by district of care for total PFAS, PFOA, or PFOS (*p* = 0.37, *p* = 0.46, and *p* = 0.93, respectively). Given the small sample size and low power, the study may be underpowered to detect true district-level differences. However, geographic analysis of total PFAS values by a more granular location of residence suggests generally increasing exposures from the coastline to the interior region, with some exceptions (Fig. 3). For example, the median total PFAS in serum was lower in participants from Paramaribo ($\tilde{x}=0.91\;\text{ng/mL}$) and Nieuw Nickerie ($\tilde{x}=1.00\;\text{ng/mL}$) compared to interior villages in the Sipaliwini district, such as Apetina/Puleowime ($\tilde{x}=1.94\;\text{ng/mL}$), Tepoe ($\tilde{x}=1.28\;\text{ng/mL}$), Alalapadi/Alalaparoe ($\tilde{x}=1.15\;\text{ng/mL}$), and Kwamalasamutu ($\tilde{x}=1.33\;\text{ng/mL}$).

Evidence of differences in serum concentration by district of care (Table 2) was revealed for PFDA (*p* = 0.04, highest median in Para) and PFUnDA (*p* < 0.01, highest median in Sipaliwini) and approached significance for PFNA and PFHxS (both *p* = 0.06, highest medians in Nickerie and Para, respectively). Tukey’s Honest Significant Difference tests conducted *a posteriori* for PFDA and PFUnDA revealed the differences were significant only between the Nickerie and Sipaliwini districts. Participants from Pikin Saron, a primarily Indigenous community in Para, had the highest median total PFAS ($\tilde{x}=2.30\;\text{ng/mL}$) of the 14 locations of residence for participants in our study (Fig. 3). While Pikin Saron is situated in Para, the community has cultures and norms that are more similar to the Indigenous participants of the southern district of Sipaliwini rather than those from the northern districts.

**Fig. 4:**
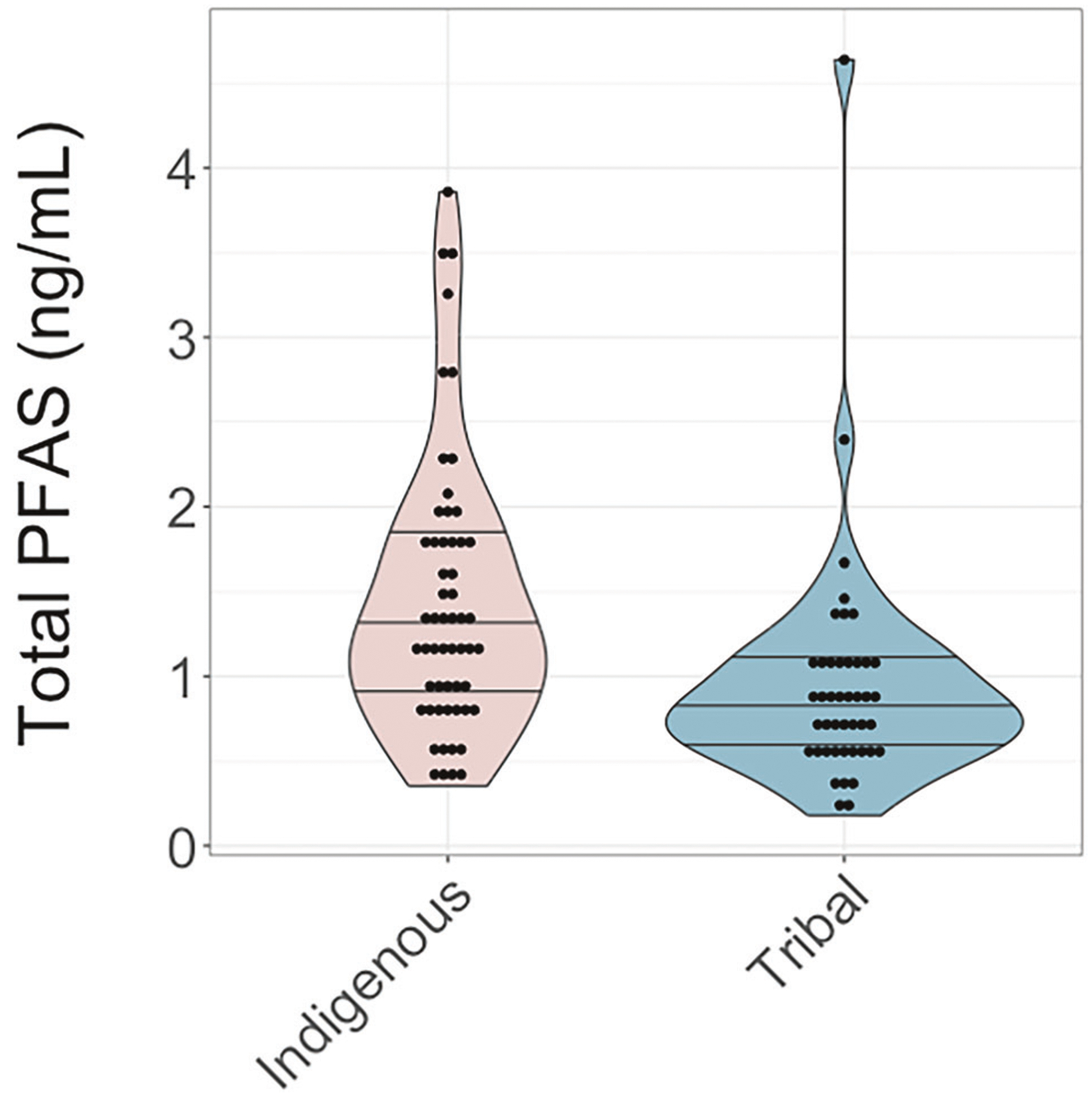
Distributions of total PFAS concentrations (ng/mL) in blood serum samples collected from Indigenous and Tribal study participants. Indigenous participants tended to have higher total PFAS levels in their blood (*n* = 56, $\tilde{x}=1.25\;\text{ng/mL}$) compared to Tribal Peoples (*n* = 44, $\tilde{x}=0.84\;\text{ng/mL}$), although the maximum detected concentration for Tribal participants (4.64 ng/mL) was higher than that for Indigenous participants (3.86 ng/mL). Using log-transformed values, a *t*-test revealed statistically significant differences between the two ethnic groups (*p* < 0.01). The lines represent the 25th, 50th, and 75th percentiles.

Compared to Tribal participants, those who identified as Indigenous tended to have significantly higher total PFAS levels in their blood ($\tilde{x}=0.84\;\text{ng/mL}$, IQR: 0.62–1.12 ng/mL for Tribal vs. $\tilde{x}=1.25\;\text{ng/mL}$, IQR: 0.87–1.79 ng/mL for Indigenous, *p* < 0.01), although the maximum detected concentration was higher in Tribal participants (4.64 ng/mL for Tribal vs. 3.86 ng/mL for Indigenous, Fig. 4). Tribal Peoples had similar median total PFAS concentrations in their blood across the districts of Sipaliwini, Para, and Paramaribo, but the maximum total PFAS concentration was substantially higher in Para participants (Supplementary Fig. 1). For Indigenous Peoples, we noted similarly elevated levels in Para compared to the other three districts (Supplementary Fig. 1). Because ethnicity was associated with location and sociodemographic factors in this study sample (Table 1), these comparisons by ethnicity cannot yet be interpreted as an independent effect, as they may instead reflect underlying differences in these potentially confounding variables.

Differences by ethnicity and district of care among individual PFAS were not always consistent with what we observed for total PFAS. For PFOS, Indigenous participants from Para (*n* = 2) had the highest median serum concentration ($\tilde{x}=1.82\;\text{ng/mL}$) compared to all other ethnicity/district groups (Supplementary Fig. 2). Indigenous participants from Sipaliwini (*n* = 35) and Nickerie (*n* = 11) had similar PFOS serum concentrations ($\tilde{x}=0.68\;\text{ng/mL}$ and $\tilde{x}=0.60\;\text{ng/mL}$, respectively) to those measured in Tribal Peoples from Para (*n* = 11) and Paramaribo (*n* = 17, $\tilde{x}=0.53\;\text{ng/mL}$ for both). Tribal women from Sipaliwini had a similar median value (*n* = 16, $\tilde{x}=0.49\;\text{ng/mL}$) to Tribal women from other districts. Indigenous individuals from Paramaribo had the second highest median concentration (*n* = 8, $\tilde{x}=0.91\;\text{ng/mL}$) but a lower maximum concentration (1.80 ng/mL) than Indigenous Peoples from Sipaliwini or Para (3.10 ng/mL for both) or Tribal women from Para (3.20 ng/mL). For PFOA, participants had similar median serum concentrations across ethnicity and districts, ranging from 0.13–0.19 ng/mL for Indigenous women and 0.12–0.15 ng/mL for Tribal women (Supplementary Fig. 3). The maximum concentration, however, was substantially higher in Indigenous participants (1.3 ng/mL) than in Tribal participants (0.28 ng/mL) for the interior Sipaliwini district. This pattern is similarly seen with PFUnDA, except that all samples from Indigenous participants in Para were non-detect (Supplementary Fig. 4).

Results from our sensitivity analysis revealed minimal differences between concentration distributions before and after imputation, with slightly lower estimates for overall median concentrations of PFOA, PFNA, PFDA, and PFUnDA and slightly higher estimates for PFHxS, PFOS, and total PFAS across the districts compared to our original analysis (Supplementary Fig. 5, Supplementary Table 1). For ANOVA tests of differences in PFNA and PFHxS concentrations by district, our original analyses approached the statistical significance level (*p* = 0.06 for both), while in analyses using multiply imputed values, there was evidence of differences by location (*p* = 0.02 and *p* = 0.03, respectively). For total PFAS, the ANOVA test was less significant (*p* = 0.48) than for the original data without imputation (*p* = 0.37). *A posteriori* Tukey’s Honest Significant Difference tests revealed one related pairwise difference that was statistically significant for each of these compounds: for PFNA, there was evidence of a difference between Para and Sipaliwini (*p* = 0.03, Para concentrations lower), and for PFHxS, there was evidence of a difference between Para and Nickerie (*p* = 0.04, Para concentrations higher).

## DISCUSSION

In this pilot study, we provide the first comprehensive assessment of PFAS exposure among pregnant women in Suriname, including participants from urban, rural, and remote populations. Contrary to our assumption that total PFAS would be highest in the blood serum of Surinamese women living in urban areas, we observed the highest levels in participants from the more isolated district of Sipaliwini, followed by the rural district of Nickerie, and lower concentrations in Para and the urban district of Paramaribo (Table 2). While the concentration of individual PFAS chemicals varied by district, Sipaliwini participants had the highest number of unique PFAS detected, contributing to the higher total PFAS measured in our study (Fig. 1). This suggests multi-source exposures in the interior.

Of the 24 PFAS compounds assessed in our analysis, 10 were detected in our study sample. Our results show universal detection of PFOS across all participants and varied detection by district and ethnicity for other PFAS. PFOS accounted for the majority of the total PFAS among all study participants, and Indigenous participants had the highest total PFAS concentrations. The prevalence of legacy PFAS, particularly PFOS, PFOA, PFNA, and PFUnDA, indicates that historical contamination, continued use of long-chain PFAS, or both were driving the blood serum PFAS concentrations measured in our study. While regional differences in median total PFAS were not statistically significant, which could be attributed to a relatively small sample size and variability within districts, our results indicate elevated concentrations in the interior and remote districts of Suriname, particularly among Indigenous participants.

Although the number of peer-reviewed publications that describe PFAS concentrations in blood samples around the globe has increased in recent years [50–53], most studies provide population reference values that are stratified by age and occasionally by sex, or provide values for different sample media, such as plasma [54]. Relatively few studies have quantified PFAS in serum samples from pregnant women, who make up our entire study population. In Brazil, Espindola Santos et al. [55] analyzed serum samples collected in 2017 from pregnant women to quantify PFOA and PFOS. Serum concentrations (geomean for PFOA: 0.51 ng/mL, geomean for PFOS: 2.06 ng/mL) were about three times higher for each of the compounds compared to our cohort in Suriname, although the women in this study were exclusively recruited during hospital visits in the highly urbanized city of Rio de Janeiro. Their detection frequency (80% for PFOA and 93% for PFOS) was similar to that presented in our study.

In a remote population of Alaska Natives, Byrne et al. [50] measured PFAS in serum samples collected from 47 women from 2013 to 2014, presenting median values and detection frequencies for PFOA (0.78 ng/mL, DF = 100%), PFNA (2.13 ng/mL, DF = 100%), PFUnDA (0.72 ng/mL, DF = 68%), and PFOS (3.35, DF = 100%). These median values are higher than those in our study, particularly for PFNA, which is consistent with other study findings [56, 57] and may be attributed to the concentrated burden of global long-range transport to polar regions.

DeLuca et al. [58] evaluated serum PFAS from 427 pregnant women in the United States from samples collected in 2009 and 2010, providing median values for PFOS (3.90 ng/mL), PFOA (1.40 ng/mL), PFNA (0.70 ng/mL), PFHxS (0.50 ng/mL), and PFDA (0.20 ng/mL), each of which are at least one order of magnitude greater than our sample medians. Our samples followed a similar pattern, with PFOS showing the highest median concentration, followed by PFOA, and generally similar concentrations of PFDA, PFNA, and PFHxS. This trend was expected, as PFOS and PFOA were historically the most commonly produced and used PFAS [3], have long half-lives [22], and have high potential for bioaccumulation in humans [59]. In some countries, such as China, PFOS and PFOA are still regularly used and are even seeing an increase in production volume [3]. Luo et al. [60] measured total PFAS concentrations in maternal serum in 234 pregnant women in China between 2018 and 2020 and found that the median value was 15.37 ng/mL, more than ten times higher than our median total PFAS of 1.05 ng/mL.

While Surinamese women appear to have lower or comparable PFAS exposures to those in more highly industrialized nations, we could find no peer-reviewed data for similar remote populations in the Amazon basin or other tropical regions, and we were therefore unable to determine whether the levels observed here are atypical for these groups. Populations in Suriname with little or no known PFAS sources and minimal access to PFAS-containing consumer products are nonetheless experiencing exposures to these contaminants. Additional analyses from the CCREOH parent study documented co-occurring exposures to other environmental contaminants, such as heavy metals, pesticides, and toxic trace elements [10, 61], raising concerns about additive or synergistic effects that may amplify adverse health outcomes beyond those attributable to PFAS alone. These findings illustrate the need for regulatory interventions that target sources, rather than relying solely on community education or point-of-exposure interventions, such as in-home drinking water filters.

### Policies and regulations

Suriname does not yet have dedicated, comprehensive, or enforceable PFAS guidelines, but PFOS is regulated under the country’s obligations to the Stockholm Convention. The Stockholm Convention is an international agreement, signed in May 2001, to reduce or eliminate the presence of POPs by decreasing or stopping their production and use in order to better protect human health globally [62]. Suriname has already created an inventory and action plan to control PFOS-containing products and contaminated sites [44, 63]. Suriname’s Environmental Framework Act (No. 97 of 2020) [64], which emphasizes the application of the Precautionary Principle [65], and Nature Conservation Act (No. 26 of 1954) [66], which focuses on the protection of wildlife and natural reserves, provide the legal basis for environmental protection and chemical management. While these do not explicitly mention PFAS, they empower the government to regulate hazardous substances and to develop and enforce national environmental protection programs. These frameworks can be expanded and enforced, and they can be used to implement PFAS-specific guidelines and regulations in the future.

In our study, PFOS and PFOA, both legacy compounds that have been phased out of production in many industrialized countries [67], accounted for the majority of the detected total PFAS across all districts and ethnic groups. This finding aligns with the Pinas et al. [44] PFAS chemical inventory, which revealed extensive storage of PFOS-containing oils used in AFFF, as well as other smaller sources like pesticides and stain-repellent carpets. PFOS was found to be the most abundant chemical in the region due to local stockpiles for its use in AFFF, primarily in the cities of Paramaribo, Nieuw Nickerie, and Zanderij (in Para).

Conversely, potential primary exposure sources in the interior communities include consumption of drinking water or local fish and wildlife from areas likely polluted by long-range atmospheric or hydrological transport [68, 69], although to our knowledge no studies have investigated exposure pathways in Suriname. Integration of our biomarker results with the regional use inventory is essential for systematic evaluation of pollution point sources and exposure pathways and can be combined with future studies that focus on environmental sampling efforts to provide a more robust exposure profile for local residents.

### Possible sources of contamination

Although our data do not provide information directly related to pollution sources, it is important to elaborate on the potential active and historical PFAS sources specific to Suriname to generate hypotheses for possible pathways of exposure. While residents in the interior may live far from urbanized areas, they are nevertheless proximal to some industries, especially mining. Alluvial gold has long been and still currently is mined in the country, as was bauxite for roughly a century [70]. Mining sites in Suriname, or in surrounding countries where environmental pollution may migrate into Suriname, are a potential source of PFAS, particularly PFOS [63]. Gold mining has led to an accelerated income for some residents, supporting the *nouveau riche* phenomenon that has been connected in other developing nations to a transformed diet, including the use of modern PFAS-containing cookware and increased consumption of packaged food over meals made with local ingredients [71]. The rural-to-urban migration, where the family lives in the surrounding rural areas while the men travel to work in the cities during the week, has also resulted in a more urban diet [72]. While urbanites may be exposed to PFAS through the burning of fossil fuels when using modern vehicles [73], traditional modes of transport, such as dugouts, may also lead to exposures through contact with contaminated waterways.

Of potential interest, Pikin Saron, the location with participants who had the highest median total PFAS concentration in our study (Fig. 3), is situated only 14 miles from Zanderij and on the same highway as the Johan Adolf Pengel International Airport. The proximity of the airport points toward a possible pathway that may contribute to exposures in Pikin Saron due to the firefighting training that takes place there and the use of AFFF. Pinas et al. [44] describe little to no waste management after firefighting training or drills. Additionally, companies that produce plastics are located near the Suriname River in the capital of Paramaribo [74], although the river flows north toward the ocean and is less likely to be the source of pollution in the interior. However, without environmental monitoring for PFAS in place, each possible source requires validation through future environmental and dietary exposure assessments.

### Disparate results

Across the country, our analyses revealed disparities among remote, rural, and urban communities. Contrary to our hypothesis, women living in the remote areas of Suriname had higher total PFAS and a greater number of detected unique PFAS compounds than those receiving care in the other three districts (Table 2). Only one serum sample had detectable levels of PFHpA, and the participant was from Sipaliwini. Participants from Para, a rural district with access to urban cities, had the highest median concentrations of PFHxS and PFDA, although the detection frequency for PFDA was the lowest among the districts; only 15% of the Para participants had detectable PFDA. Women from Nickerie, a generally agricultural district that also contains the second largest city in Suriname, had the highest median concentration for PFOS, though the differences among districts were not significant (*p* = 0.93). Participants from Nickerie also had the highest median concentration for PFNA, which was detected in 100% of the samples from this district. Paramaribo did not have the highest median concentration for any individual PFAS and only had the highest detection frequency for PFHxS (80%).

Women from Sipaliwini also tended to have a higher PFUnDA serum concentration and were more likely to have detectable levels of PFUnDA (DF = 94%) compared to the other districts (Table 2). PFUnDA, a byproduct of the deterioration of anti-grease and anti-stain coatings on carpet, couches, and food packaging [75], can bioaccumulate and bioconcentrate [76], making consumption of fish and other aquatic animals a possible route of elevated exposure. Byrne et al. [50] similarly noted PFUnDA as among the most frequently detected PFAS in their study population of remote Alaska Natives, who are also subsistence fishermen.

Within our sample, possible ethnic disparities also emerged. Indigenous Peoples exhibited higher median values for total PFAS than those from Tribal Peoples, although the overall maximum concentration of total PFAS came from a sample corresponding to a woman who identified as Tribal (Fig. 4). Differences could not be confirmed as directly associated with ethnicity due to potential confounding by other factors, such as education, income, or district of residence. These disparities varied by district and by PFAS compound. Indigenous participants from Para had the highest median PFOS concentrations. They were, however, entirely non-detects for PFUnDA, unlike those from the other three districts. Tribal participants showed relatively consistent concentrations across districts, except for elevated values in Para.

### Study strengths

Through this preliminary work defining exposure profiles for pregnant women in Suriname, our research team has begun to address a critical knowledge gap for a vulnerable population in an understudied region. The inclusion of isolated and remote communities, which are typically omitted from health research due to logistical challenges, provides a rare insight into PFAS exposures for populations living in relatively pristine rainforest environments, far from urbanized cityscapes. Our analysis quantified both long- and short-chain PFAS chemicals, allowing us to more fully characterize the exposure profile of our study sample and to consider potential sources of contamination. To our knowledge, this is the first study examining PFAS exposures in pregnant women in Suriname.

For this evaluation, we used previously collected samples from the CCREOH parent study, which additionally collected multiple biological samples (e.g., blood, urine, hair) for quantification of heavy metals, pesticides, and other environmental contaminants [10]. This will allow us to integrate our findings related to blood serum PFAS with prior and future analyses of this same study population and to assess exposures to chemical mixtures and evaluate cumulative exposures and health risks. Furthermore, our analysis provides high-quality exposure data that are suitable for quantitative risk assessments and comparisons with other populations.

### Study limitations

Our serum samples were purposively selected from the CCREOH study based on our research question and do not represent a random sample. Had we selected different samples, our results could have differed enough to affect our interpretation and conclusions. The cross-sectional nature of the PFAS measurements, with samples taken from a single timepoint during pregnancy, cannot capture temporal variability in exposure, nor can we make any claims about exposure risks throughout gestation. While the larger parent study design was longitudinal and included follow-up assessments of the children for neurodevelopmental and other health outcomes, we did not establish relationships between possibly dynamic PFAS exposures and health outcomes measured at later timepoints.

All of our study participants identified as Indigenous or Tribal Peoples in order to reduce differences related to the risk of PFAS exposure, such as culture-based diet and behavioral norms. However, because of this, we are unable to compare their serum PFAS concentrations to those of other demographic groups in the country. Additionally, we selected 50% of our study samples from women living in the interior district of Sipaliwini, purposefully oversampling Indigenous women relative to the proportion of Indigenous Peoples in the total Surinamese population. This stratified, non-random sampling method enabled us to gather sufficient data from this under-represented group for which data are highly limited, which can be used to guide future research in the region. Unfortunately, this disproportionate allocation simultaneously precludes the generalizability of our results to the greater Surinamese population. A larger, more inclusive assessment could address the question of differences among Indigenous Peoples, Tribal Peoples, and other demographic groups.

As is the case for many environmental health studies, our relatively small sample size (*n* = 100) could have limited our statistical power to detect regional and demographic differences and prevented additional subgroup analyses. Low power increases the chances of false-negative results; however, this study was exploratory in nature and therefore more appropriate for generating preliminary evidence and informing future research. For example, we were unable to disentangle differences in serum PFAS concentrations by ethnicity from potential confounding by geography or socioeconomic status. Our efforts should guide the development of larger studies with greater capacity for robust analyses, such as multiple regressions or multivariate analyses that can include covariates to help isolate individual effects, assess potential confounders, and provide more conclusive results.

Our analyses included only detectable concentrations of PFAS compounds, which may have impacted our ability to accurately estimate concentrations. For individual PFAS compounds, we likely overestimated average concentrations by excluding non-detects. For total PFAS concentrations, this may have led to underestimating the total concentration since non-detect does not always equate to true zero concentrations. To address these concerns, we conducted a sensitivity analysis using multiple imputations by chained equations to impute non-detect values for individual PFAS compounds with low missingness (<30% missing observations). These analyses using the multiply imputed datasets led to similar conclusions as the original analyses.

Finally, while our analytical methods encompassed 24 unique PFAS compounds, this represents only a small fraction of the thousands of PFAS that have been used since their inception. PFAS not included in our panels may nevertheless be present throughout Suriname, contributing to overall body burden and potentially challenging the interpretations made in our study if those distributions follow patterns inconsistent with those discussed here. The lack of environmental media samples, such as water, soil, air, and food, prevented us from making direct connections between environmental contamination levels and the PFAS biomarker concentrations in our study.

### Future research

Expansion of biomonitoring efforts to include men, children, and non-pregnant women from ethnic groups beyond Indigenous and Tribal Peoples would provide a more comprehensive characterization of population-level exposures and enable identification of other vulnerable subgroups. While the participants included in our study may accurately represent their demographic and geographic groups, future studies should use random sampling methods to adequately verify their representativeness and improve generalizability. Additionally, repeated biomonitoring over time would allow participants and researchers to document temporal trends in PFAS exposures, track the emergence of new PFAS replacement compounds, and assess the effectiveness of any implemented interventions. Given the changes to PFAS regulations in other countries [67], analysis of additional biological samples would provide data for comparison with our samples, which were collected between 2016 and 2019 and may not represent current exposures. Efforts should especially focus on evaluating the biological relevance of differences in PFAS concentrations among distinct groups.

In order to identify sources of pollution, environmental sampling should be conducted across the country, especially in riparian areas where surface water and groundwater transport of PFAS may be occurring. Our list of possible sources of contamination presented here can help guide an environmental sampling plan. Similar to biomonitoring, environmental monitoring should include longitudinal data collection across a range of geographical locations in order to better characterize the fate and transport of PFAS in the region. Spatial autocorrelation analyses (e.g., Moran’s I) can help identify clusters of contamination, which can allow researchers to narrow in on possible “hot spots” for further evaluation. In combination, these data can be used to track contaminant movement based on geophysical and chemical properties of the surrounding environment, as well as local changes over time. Paired environmental and biological sampling techniques would be valuable for identifying exposure pathways and informing targeted intervention strategies.

Finally, relevant health data in the country are limited, with few published studies that address population-level health outcomes. Cardiovascular health is a priority for the country, with recent initiatives to expand resources for hypertension and diabetes care [77–79]. An evaluation of the 2017 State Health Foundation revealed that the highest rates of prescription medication use were for cardiovascular diseases and respiratory disorders, and that the highest rates were for residents living in the rural-coastal regions, followed by urban-coastal residents and finally by those living in the rural-interior [80], which may reflect a lack of access. Future PFAS assessments should consider using these three categories (i.e., rural-coastal, urban-coastal, and rural-interior) to describe the regions and people of Suriname. The Pan American Health Organization (PAHO) 2024 annual public health report [79] demonstrates official efforts to improve healthcare access in remote regions, to augment available health data through the development and employment of a new digitized mortality surveillance system, and to enhance perinatal health and well-being. Efforts should be made to incorporate environmental health into both outreach and data capture initiatives as a venue for health promotion and preventative interventions.

## CONCLUSION

In our study sample of pregnant Surinamese women, remote interior communities experienced higher total PFAS exposures than urban populations. This suggests a departure from exposure patterns documented in other areas, where PFAS biomarkers often correlate with urbanization and proximity to industrial sites that manufacture or use products that contain these compounds. This seemingly inverted exposure gradient indicates that factors other than urbanization or consumer product use may be driving PFAS exposures in Suriname.

Although numerical differences in serum concentrations were observed, many were not statistically significant, and those that were may reflect sampling variability rather than true differences due to the limited sample size and low statistical power of this study. Additionally, our non-random sample of Indigenous and Tribal women is not representative of the general Surinamese population. Because this was an exploratory study, we are unable to draw precise conclusions regarding exposures across Suriname. We recommend extended biomonitoring efforts, as well as direct investigations into potential sources of pollution and pathways of exposure, including those specific to interior residents, Indigenous communities, and Tribal Peoples.

Enforcement of regulations for PFAS manufacturing, use, and disposal is currently lacking in Suriname. The establishment of drinking water standards, food safety thresholds, and reference values for biomonitoring efforts, ideally tailored to the various subpopulations in Suriname, would provide invaluable tools for environmental protection and health promotion through risk prevention. International guidelines, while informative, may not adequately account for the unique exposure patterns, cultural and behavioral norms, dietary needs, and other susceptibility factors specific to Surinamese populations. Implementation of frameworks like the Stockholm Convention, informed by these local exposure data, can enhance the effectiveness and adoption of public health interventions.

## Supplementary Material

Supplemental Figure 1

Supplemental Figure 2

Supplemental Figure 3

Supplemental Figure 4

Supplemental Figure Captions

Supplemental Figure 5

Supplemental Table 1

The online version contains supplementary material available at https://doi.org/10.1038/s41370-026-00950-y.

## Figures and Tables

**Table 1.** Participant demographics.

	All women	Indigenous women	Tribal women	
	*n* (%)			*X*^2^ *p*-value

**Total**	100 (100)	56 (56)	44 (44)	

**District**				**<0.01** ^a^

Paramaribo	25 (25)	8 (14)	17 (39)	

Nickerie	11 (11)	11 (20)	0 (0)	

Para	13 (13)	2 (4)	11 (25)	

Sipaliwini	51 (51)	35 (63)	16 (36)	

**Parity**				0.35

0	31 (31)	20 (36)	11 (25)	

1	17 (17)	8 (14)	9 (20)	

2	14 (14)	9 (16)	5 (11)	

3	17 (17)	8 (14)	9 (20)	

4	9 (9)	3 (5)	6 (14)	

5+	11 (11)	7 (13)	4 (9)	

Not answered	1 (1)	1 (2)	0 (0)	

**Education**				**<0.01** ^a^

No education	11 (11)	7 (13)	4 (9)	

Primary	34 (34)	26 (46)	8 (18)	

Lower vocational or lower secondary	38 (38)	19 (34)	19 (43)	

Upper vocational or upper secondary	12 (12)	3 (5)	9 (20)	

Tertiary	4 (4)	0 (0)	4 (9)	

Not answered	1 (1)	1 (2)	0 (0)	

**Monthly household income (SRD)**				0.13

<1500	62 (62)	41 (73)	21 (48)	

1500–2999	18 (18)	8 (14)	10 (23)	

3000–4999	7 (7)	2 (4)	5 (11)	

5000+	6 (6)	3 (5)	3 (7)	

Not answered	7 (7)	2 (4)	5 (11)	

	$\bar{x}\;(\mathrm{SD})$			t-test *p*-value

**Age (years)**	26.0 (6.6)	25.6 (7.0)	26.6 (6.2)	0.46

Percentages may not add to 100 due to rounding.

$\bar{x}$: Arithmetic mean.

SD: Arithmetic standard deviation.

a Statistically significant at α ≤ 0.05.

**Table 2.** Blood serum PFAS concentrations (ng/mL) for pregnant Surinamese women by district.

	All Districts		Paramaribo		Nickerie		Para		Sipaliwini		ANOVA^a^ *p*-value
	*n* = 100		*n* = 25		*n* = 11		*n* = 13		*n* = 51		
	%DF	Median (IQR)	%DF	Median (IQR)	%DF	Median (IQR)	%DF	Median (IQR)	%DF	Median (IQR)	
Total PFAS	-	1.05 (0.73–1.45)	-	0.93 (0.61–1.44)	-	1.00 (0.79–1.17)	-	0.78 (0.52–1.30)	-	1.12 (0.84–1.69)	0.37
PFHpA	1	0.09 (0.09–0.09)	0	ND	0	ND	0	ND	2	0.09 (0.09–0.09)	-
PFOA	86	0.15 (0.11–0.22)	92	0.15 (0.09–0.20)	82	0.14 (0.12–0.16)	92	0.15 (0.12–0.18)	82	0.15 (0.11–0.28)	0.46
PFNA	84	0.09 (0.07–0.13)	80	0.08 (0.06–0.11)	100	0.11 (0.07–0.11)	77	0.08 (0.06–0.11)	84	0.10 (0.07–0.15)	0.06
PFDA	56	0.08 (0.06–0.09)	28	0.06 (0.06–0.07)	73	0.06 (0.06–0.06)	15	0.09 (0.09–0.09)	76	0.08 (0.07–0.10)	**0.04** ^b^
PFUnDA	72	0.09 (0.07–0.12)	40	0.08 (0.06–0.09)	82	0.07 (0.06–0.08)	38	0.09 (0.06–0.10)	94	0.11 (0.08–0.13)	**<0.01** ^b^
PFDoDA	1	0.10 (0.10–0.10)	0	ND	0	ND	0	ND	2	0.10 (0.10–0.10)	-
PFHxS	71	0.06 (0.04–0.10)	80	0.07 (0.04–0.10)	55	0.04 (0.03–0.04)	77	0.09 (0.05–0.12)	69	0.06 (0.04–0.09)	0.06
PFHpS	3	0.06 (0.06–0.06)	0	ND	0	ND	8	0.06 (0.06–0.06)	4	0.06 (0.06–0.06)	-
PFOS	100	0.59 (0.45–0.84)	100	0.57 (0.45–0.82)	100	0.60 (0.55–0.71)	100	0.53 (0.39–0.87)	100	0.59 (0.46–0.85)	0.93
NMeFOSAA	1	0.65 (0.65–0.65)	0	ND	0	ND	8	0.65 (0.65–0.65)	0	ND	-

DF: Detection Frequency (number of samples with detectable PFAS concentrations out of the 100 total study samples).

IQR: Interquartile Range (25th–75th percentiles).

a Analysis of variance.

b Statistically significant at *α* ≤ 0.05.

Samples were collected from December 2016 to July 2019. From each of the samples (n=100), we quantified 24 distinct PFAS chemicals as part of the National Institutes of Environmental Health Science Human Health Exposure Analysis Resource (HHEAR) program, and 10 were detected in our study samples. ANOVA tests for differences among the districts were conducted after log-transforming the serum concentration data to normalize the distributions.

## Data Availability

De-identified data and code used for data analyses are available upon request.
