# Supplementary material for: Per- and polyfluoroalkyl substances (PFAS) exposure profiles for pregnant women living in urban, rural, and remote areas of Suriname, South America suggest higher serum concentrations in remote rainforest communities
Source: J Expo Sci Environ Epidemiol. Author manuscript; Available in PMC 2026 Aug 17. (PMC13479382; doi:10.1038/s41370-026-00950-y)
Supplement: Supplementary file 1 [file NIHMS2198727-supplement-Supplemental_Figure_Captions.docx]

**Supplemental Figure 1: Distributions of total PFAS concentrations (ng/mL) in blood serum samples by ethnicity and by district.** Median values for Indigenous women were similar across Paramaribo (n=8, $\tilde{x}$=1.38 ng/mL), Sipaliwini (n=35, $\tilde{x}$=1.36 ng/mL), and Nickerie (n=11, $\tilde{x}$=1.00 ng/mL). Indigenous participants from the Para region had the highest median (n=2, $\tilde{x}$=2.30 ng/mL) and maximum (3.86 ng/mL) concentrations. Median values for Tribal women were similar across regions (Paramaribo n=17 and $\tilde{x}$=0.83 ng/mL, Para n=11 and $\tilde{x}$=0.78, Sipaliwini n=16 and $\tilde{x}$=0.85), although participants from the Para region had a much higher maximum concentration (4.64 ng/mL).

**Supplemental Figure 2: Distributions of PFOS concentrations (ng/mL) in blood serum samples collected from study participants by district and by ethnicity.** Median values for Indigenous women were lowest in Nickerie (n=11, $\tilde{x}$=0.60 ng/mL), followed by Sipaliwini (n=35, $\tilde{x}$=0.68 ng/mL), Paramaribo (n=8, $\tilde{x}$=0.91 ng/mL), and highest in the Para district (n=2, $\tilde{x}$=1.82 ng/mL). Median values for Tribal women were similar across regions (Paramaribo n=17 and $\tilde{x}$=0.53 ng/mL, Para n=11 and $\tilde{x}$=0.53, Sipaliwini n=16 and $\tilde{x}$=0.49), although participants from the Para region had a much higher maximum concentration (3.20 ng/mL).

**Supplemental Figure 3: Distributions of PFOA concentrations (ng/mL) in blood serum samples collected from study participants by district and by ethnicity.** Median values for Indigenous women were similar across districts, with the lowest value in Paramaribo (n=8, $\tilde{x}$=0.13 ng/mL), followed by Nickerie (n=9, $\tilde{x}$=0.14 ng/mL), Sipaliwini (n=28, $\tilde{x}$=0.17 ng/mL), and highest in the Para district (n=2, $\tilde{x}$=0.19 ng/mL). Median values for Tribal women were similar across regions (Paramaribo n=15 and $\tilde{x}$=0.15 ng/mL, Para n=10 and $\tilde{x}$=0.15, Sipaliwini n=14 $\tilde{x}$=0.12). An Indigenous participant from Sipaliwini had the highest maximum concentration (1.30 ng/mL).

**Supplemental Figure 4: Distributions of PFUnDA concentrations (ng/mL) in blood serum samples collected from study participants by district and by ethnicity.** Median values for Indigenous women varied across districts, with the lowest value in Nickerie (n=9, $\tilde{x}$=0.07 ng/mL), followed by Paramaribo (n=5, $\tilde{x}$=0.09 ng/mL), Para (n=1, $\tilde{x}$=0.10 ng/mL) and highest in the Sipaliwini district (n=35, $\tilde{x}$=0.19 ng/mL). Median values for Tribal women were similar across regions (Paramaribo n=5 and $\tilde{x}$=0.07 ng/mL, Para n=4 and $\tilde{x}$=0.08, Sipaliwini n=13 and $\tilde{x}$=0.08). An Indigenous participant from Sipaliwini had the highest maximum concentration (0.38 ng/mL).

**Supplemental Figure 5: Distributions of log-transformed PFAS data A) before imputation and B) after imputation.** Non-detects were replace using multiple imputations by chained equations. Our sensitivity analysis revealed minimal differences among the distributions.
