# Supplementary material for: Per- and polyfluoroalkyl substances (PFAS) exposure profiles for pregnant women living in urban, rural, and remote areas of Suriname, South America suggest higher serum concentrations in remote rainforest communities
Source: J Expo Sci Environ Epidemiol. Author manuscript; Available in PMC 2026 Aug 17. (PMC13479382; doi:10.1038/s41370-026-00950-y)
Supplement: Supplementary file 2 [file NIHMS2198727-supplement-Supplemental_Table_1.docx]

**Supplemental Table 1: Results of sensitivity analysis for data presented in Table 2 using imputed data for PFAS (ng/mL) with<30% non-detects.** Data were multiply imputed using multiple imputations by chained equations.

|  | All Districts | | Paramaribo | | Nickerie | | Para | | Sipaliwini | |  |
| --- | --- | --- | --- | --- | --- | --- | --- | --- | --- | --- | --- |
|  | n = 100 | | n = 25 | | n = 11 | | n = 13 | | n = 51 | |  |
|  | % DF | Median (IQR) | % DF | Median(IQR) | % DF | Median(IQR) | % DF | Median(IQR) | % DF | Median(IQR) | ANOVA^‡^  p-value |
| Total PFAS | - | 1.16 (0.85-1.48) | *-* | 1.07 (0.79-1.44) | *-* | 1.03 (0.88-1.23) | *-* | 0.95 (0.71-1.38) | - | 1.20 (0.91-1.69) | 0.48 |
| PFOA | 86 | 0.13 (0.10-0.19) | 92 | 0.13 (0.11-0.19) | 82 | 0.13 (0.09-0.16) | 92 | 0.15 (0.12-0.17) | 82 | 0.12 (0.10-0.21) | 0.50 |
| PFNA | 84 | 0.09 (0.06-0.12) | 80 | 0.08 (0.06-0.10) | 100 | 0.11 (0.07-0.11) | 77 | 0.06 (0.05-0.09) | 84 | 0.10 (0.07-0.14) | **0.02*** |
| PFDA | 56 | 0.06 (0.06-0.08) | 28 | 0.06 (0.05-0.06) | 73 | 0.06 (0.06-0.06) | 15 | 0.06 (0.06-0.07) | 76 | 0.08 (0.06-0.9) | **< 0.01*** |
| PFUnDA | 72 | 0.08 (0.06-0.11) | 40 | 0.07 (0.06-0.09) | 82 | 0.07 (0.06-0.09) | 38 | 0.06 (0.06-0.08) | 94 | 0.11 (0.08-0.13) | **< 0.01*** |
| PFHxS | 71 | 0.06 (0.04-0.09) | 80 | 0.07 (0.04-0.09) | 55 | 0.04 (0.03-0.06) | 77 | 0.09 (0.05-0.11) | 69 | 0.06 (0.04-0.08) | **0.03*** |
| PFOS | 100 | 0.59 (0.45-0.84) | 100 | 0.57 (0.45-0.82) | 100 | 0.60 (0.55-0.71) | 100 | 0.53 (0.39-0.87) | 100 | 0.59 (0.46-0.85) | 0.93 |

DF: Detection Frequency (number of samples with detectable PFAS concentrations out of the 100 total study samples)

IQR: Interquartile Range (25^th^ - 75^th^ percentiles)

ANOVA^‡^: Analysis of Variance, conducted on log-transformed serum concentrations

*: Statistically significant at $\alpha\leq$ 0.05
